# Reversible inactivation of a peptidoglycan transpeptidase by a β-lactam antibiotic mediated by β-lactam-ring recyclization in the enzyme active site

**DOI:** 10.1038/s41598-017-09341-8

**Published:** 2017-08-22

**Authors:** Zainab Edoo, Michel Arthur, Jean-Emmanuel Hugonnet

**Affiliations:** INSERM UMRS 1138, Sorbonne Universités, UPMC Univ Paris 06; Sorbonne Paris Cité, Université Paris Descartes, Université Paris Diderot; Centre de Recherche des Cordeliers, 75006 Paris, France

## Abstract

β-lactam antibiotics act as suicide substrates of transpeptidases responsible for the last cross-linking step of peptidoglycan synthesis in the bacterial cell wall. Nucleophilic attack of the β-lactam carbonyl by the catalytic residue (Ser or Cys) of transpeptidases results in the opening of the β-lactam ring and in the formation of a stable acyl-enzyme. The acylation reaction is considered as irreversible due to the strain of the β-lactam ring. In contradiction with this widely accepted but poorly demonstrated premise, we show here that the acylation of the L,D-transpeptidase Ldt_fm_ from *Enterococcus faecium* by the β-lactam nitrocefin is reversible, leading to limited antibacterial activity. Experimentally, two independent methods based on spectrophotometry and mass spectrometry provided evidence that recyclization of the β-lactam ring within the active site of Ldt_fm_ regenerates native nitrocefin. Ring strain is therefore not sufficient to account for irreversible acylation of peptidoglycan transpeptidases observed for most β-lactam antibiotics.

## Introduction

β-lactams remain the corner stone of antibacterial chemotherapy 76 years after the first therapeutic use of an antibiotic, namely penicillin, in 1941. β-lactams kill bacteria by inactivating the D,D-transpeptidases responsible for the last cross-linking step of peptidoglycan synthesis^1^. The latter cell wall polymer is a giant macromolecule of 10^9^ to 10^10^ daltons that completely surrounds bacterial cells and plays an essential role in counteracting the osmotic pressure of the cytoplasm during the entire cell cycle including cell division^2^. β-lactams are structure analogues of the peptidoglycan precursors and act as suicide substrates of the transpeptidases^3^. The enzymes catalyze formation of peptidoglycan cross-links in a two-step reaction^4^. In the first step, the D,D-transpeptidases interact with an acyl donor containing a pentapeptide stem ending in D-Ala^4^-D-Ala^5^, leading to the release of D-Ala^5^ and to the formation of an ester bond between the carbonyl of D-Ala^4^ and the catalytic Ser residue of the enzymes. In the second step, nucleophilic attack of the resulting acyl-enzyme by the acyl acceptor generates the peptidoglycan cross-link. Similar to the first step of the transpeptidation reaction, nucleophilic attack of the carbon carbonyl of β-lactams by the catalytic Ser leads to rupture of the amide bond of the β-lactam ring and inactivation of the D,D-transpeptidases. The resulting acyl-enzymes are highly stable, with typical half-lives in the order of several hours, due to low hydrolysis rates. For this reason, the inactivation reaction is considered as irreversible since recovery of enzyme activity through hydrolysis of the drug-enzyme adduct occurs at a timescale that is too large to compromise antibacterial activity.

**Figure 1:**
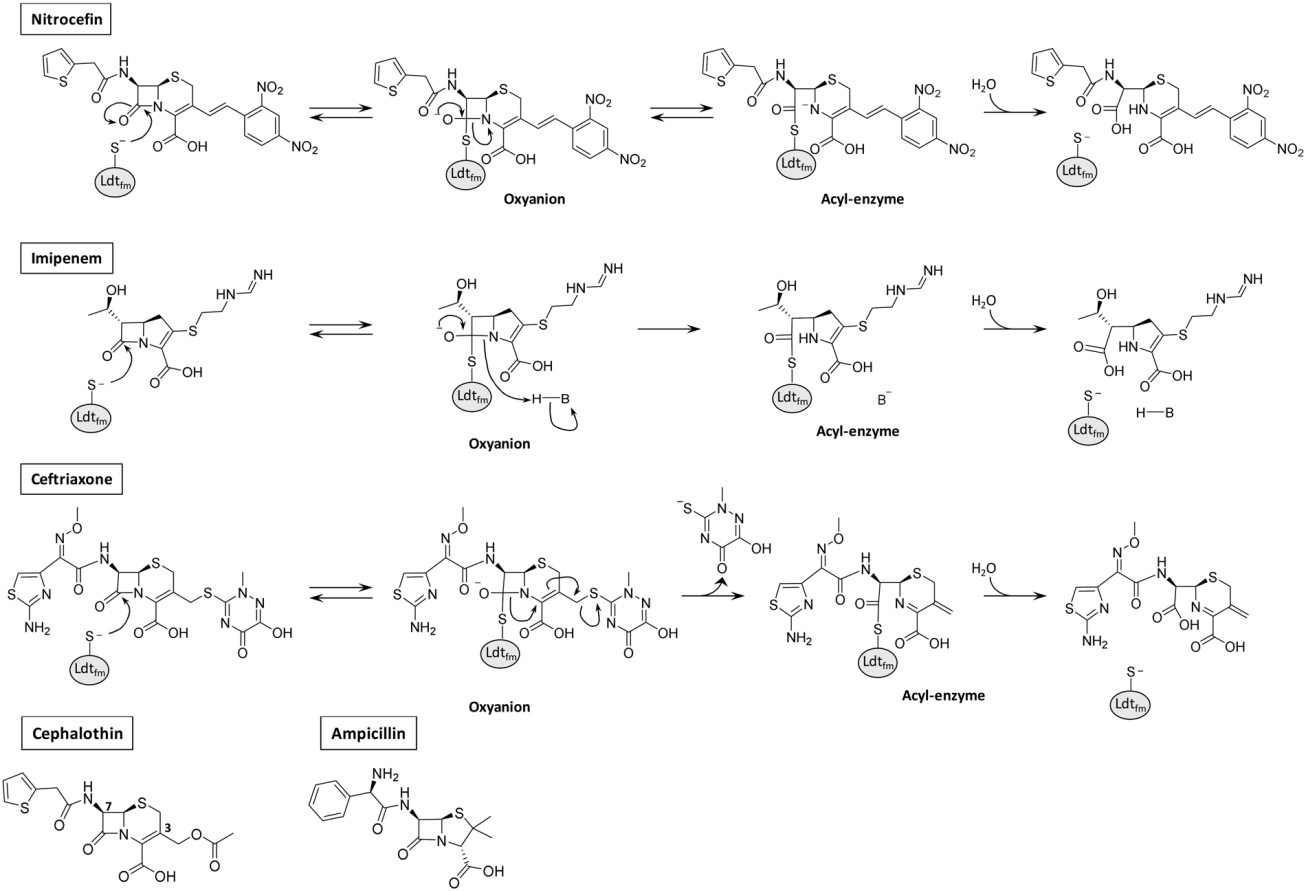
Reactions catalyzed by the L,D-transpeptidase Ldt_fm_ with nitrocefin, imipenem, and ceftriaxone. The figure also shows the structures of cephalothin and ampicillin. The base (B) that provides a proton to the β-lactam nitrogen of imipenem has not been identified^23, 26^.

The D,D-transpeptidases have been historically referred to as penicillin-binding proteins (PBPs) since these enzymes were routinely identified by covalent labeling with radioactive penicillin followed by gel electrophoresis^1^. PBPs belong to a super family of mechanistically and structurally related active-site serine enzymes that also comprise D,D-carboxypeptidases, endopeptidases, and most β-lactamases responsible for β-lactam resistance by drug detoxification^3, 5^. The acyl-enzymes formed by the acylation of the Ser residue of β-lactamases are rapidly hydrolyzed leading to turnover and drug detoxification. More recently, the family of β-lactam-interacting enzymes has been enriched by the detection of active-site cysteine L,D-transpeptidases that by-pass the D,D-transpeptidase activity of PBPs in β-lactam-resistant mutants of *Enterococcus faecium* and *Escherichia coli* ^6, 7^. These L,D-transpeptidases were also found to be the main peptidoglycan cross-linking enzymes in *Clostridium difficile* and in mycobacteria^8^. The L,D-transpeptidases are structurally unrelated to active-site Ser PBPs and cleave the L-Lys^3^-D-Ala^4^ or diaminopymelate^3^-D-Ala^4^ peptide bond of an acyl donor containing a tetrapeptide stem^9, 10^. In spite of this difference in substrate specificity, L,D-transpeptidases interact with β-lactams belonging to the carbapenem class^11^ (Fig. 1) that are currently being evaluated for the treatment of tuberculosis^12^ and of pulmonary infections due to *Mycobacterium abscessus* in cystic fibrosis patients^13^. The L,D-transpeptidases are also acylated by β-lactams belonging to the cephalosporin and penam classes but this does not lead to target inactivation and antibacterial activity since the corresponding adducts are prone to hydrolysis^14^.

Due to the strain of the four-membered ring of β-lactams, the acylation reaction is considered as irreversible in the sense that re-sealing of the scissile amide bond of the β-lactam ring is not thought to occur at any significant rate^15, 16^. In this study, we have critically evaluated this postulate by studying the acylation of the L,D-transpeptidase Ldt_fm_ from *E*. *faecium* by nitrocefin, a chromogenic cephalosporin. We show that this drug-enzyme combination displays high acylation and low hydrolysis rates leading to acyl-enzyme accumulation. This offered the possibility to assay for recyclization of the β-lactam ring by two approaches based on the use of a competing β-lactam or a competing enzyme. We provide evidence that recyclization of the β-lactam ring of nitrocefin in the acyl-enzyme regenerates the native drug that can freely diffuse out of the Ldt_fm_ active site. These data indicate that the irreversible nature of the acylation reaction is not exclusively driven by the strain of the four-membered ring of β-lactams.

## Results

### Modification of the absorbance spectrum of nitrocefin following acylation of the L,D-transpeptidase Ldt_fm_

**Figure 2:**
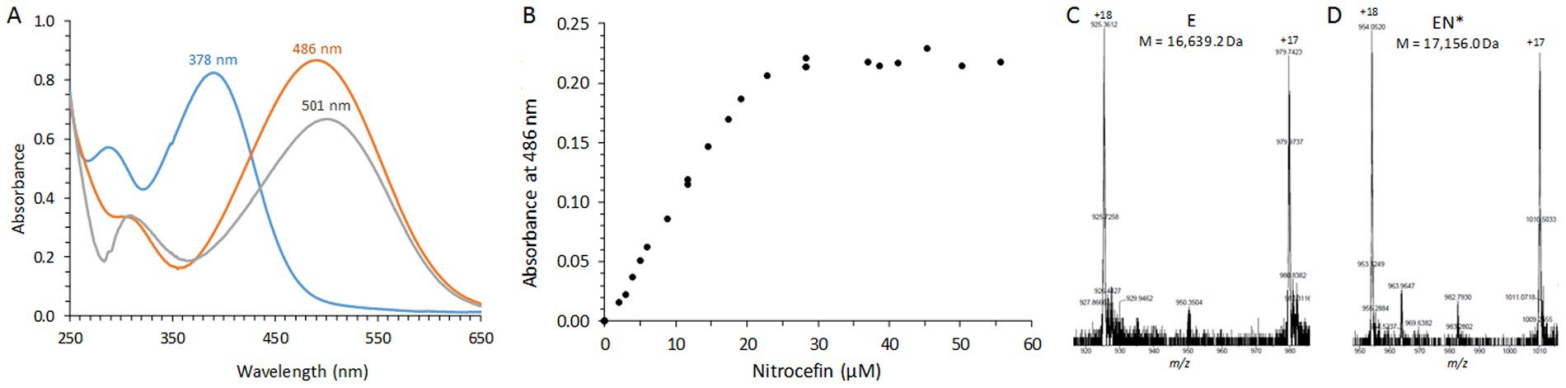
Determination by spectrophotometry of the acylation of Ldt_fm_ by nitrocefin. (**A**) Absorbance spectra of various forms of nitrocefin (50 µM). Blue, native nitrocefin; orange, nitrocefin hydrolyzed by BlaC; grey, nitrocefin in the acyl-enzyme formed with Ldt_fm_. (**B)** Titration of the active site of Ldt_fm_ (22 µM) by nitrocefin. (**C** and **D**) Mass spectra of Ldt_fm_ and of the acyl-enzyme formed with nitrocefin. The average mass of Ldt_fm_ (E) and of the acyl-enzyme (EN*) were deduced from the *m*/*z* ratios of the [M + 17 H]^17+^ and [M + 18 H]^18+^ ions. The calculated mass of nitrocefin, Ldt_fm_, and of the acyl-enzyme were 516.5 Da, 16,639.3 Da, and 17,155.8 Da, respectively.

Hydrolysis of the β-lactam ring of nitrocefin (50 µM) by the β-lactamase BlaC (1 µM) in 100 mM sodium phosphate buffer (pH 6.0) produced the expected large increase in the absorbance at 486 nm (Fig. 2A). Under these conditions, the variation in the molar extinction coefficient (Δε) was 15,200 M^−1^ cm^−1^ (1,400 M^−1^ cm^−1^ *versus* 16,600 M^−1^ cm^−1^ for native and hydrolyzed nitrocefin, respectively). Incubation of Ldt_fm_ (22 µM) with increasing concentrations of nitrocefin (0 to 56 µM) also led to an increase of the absorbance at 486 nm (Fig. 2B). The increase in the absorbance was linear until the concentration of nitrocefin exceeded that of the enzyme. This result indicates that the increase in the absorbance at 486 nm is due to the rupture of the β-lactam ring of nitrocefin upon acylation of the catalytic Cys residue of Ldt_fm_. In agreement, a covalent adduct was detected by mass spectrometry (Fig. 2C). The spectra of nitrocefin hydrolyzed by BlaC and of nitrocefin linked to Ldt_fm_ in the acyl-enzyme were similar with two minor differences. Nitrocefin in the acyl-enzyme absorbed less than hydrolyzed nitrocefin (ε = 9,900 M^−1^ cm^−1^ *versus* 16,600 M^−1^ cm^−1^ at 486 nm, respectively). A minor red shift might occur in the absorbance maximum (λ_max_ of 501 nm *versus* 486 nm, respectively). Thus, nitrocefin provides a sensitive assay to titrate the active site of Ldt_fm_.

### Kinetic analyses of Ldt_fm_ acylation by nitrocefin

**Figure 3:**
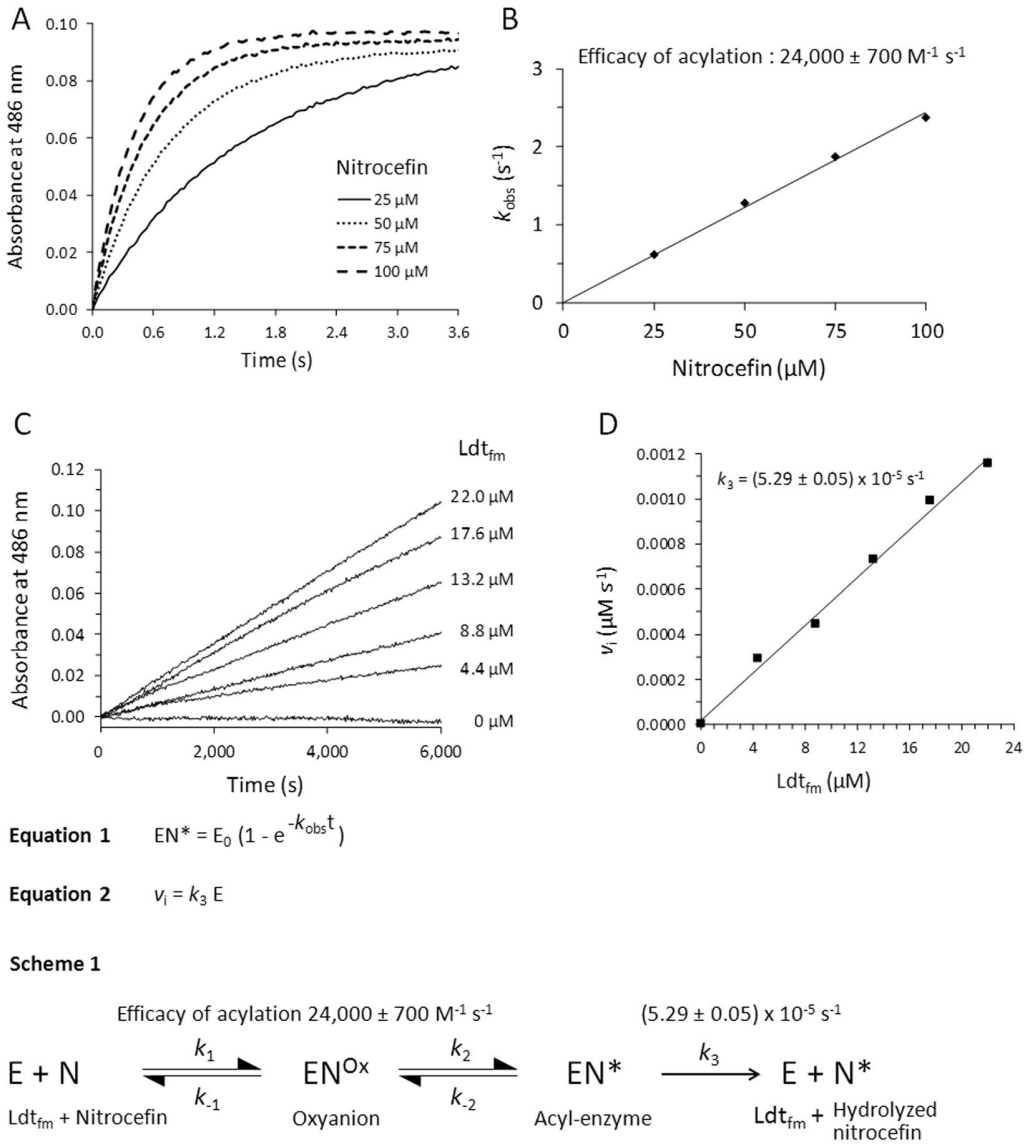
Kinetics of Ldt_fm_ acylation by nitrocefin and of hydrolysis of the resulting acyl-enzyme. (**A)** Stopped-flow kinetics of acylation of Ldt_fm_ (11 µM) by nitrocefin (25, 50, 75, and 100 µM) in 100 mM sodium phosphate buffer (pH 6.0) at 20 °C. The value of *k*_obs_ was determined by fitting Equation 1 to data. E_0_, concentration of native Ldt_fm_ at t = 0. (**B)** Variation of *k*_obs_ as a function of the concentration of nitrocefin. (**C)** Hydrolysis of a fixed concentration of nitrocefin (50 µM) by Ldt_fm_ at various concentrations (0 to 22 µM). (**D)** The initial rate of hydrolysis (*v*_i_) was plotted as a function of the concentration of Ldt_fm_ and the value of *k*_3_ was determined by linear regression (Equation 2) considering that Ldt_fm_ is fully acylated under these conditions.

Stopped-flow kinetics were performed to assess the efficacy of acylation of Ldt_fm_ (11 µM) by nitrocefin (25, 50, 75, and 100 µM; Fig. 3A). The concentration of the acyl-enzyme (EI*) was determined based on the difference in the molar extinction coefficient (Δε_486 nm_ = 8,500 M^−1^ cm^−1^) between native nitrocefin (ε = 1,400 M^−1^ cm^−1^) and nitrocefin in the acyl-enzyme (ε = 9,900 M^−1^ cm^−1^) (Fig. 2). To determine the value of *k*_obs_, as defined in equation 1 (Fig. 3), exponential rise to maximum was fitted to kinetic data. Full acylation of Ldt_fm_ was observed at all the concentrations of nitrocefin that were tested and the value of *k*_obs_ increased linearly with the concentration of nitrocefin (Fig. 3B). The slope provided an estimate of the efficacy of the acylation reaction (24,000 ± 700 M^−1^ s^−1^).

### Hydrolysis of the acylenzyme

Incubation of nitrocefin (50 µM) with various concentrations of Ldt_fm_ (0 to 22 µM) for an extended time period (14 hours) revealed an increase in the absorbance at 486 nm (Fig. 3C), which was proportional to the enzyme concentration (Fig. 3D). The concentration of hydrolyzed nitrocefin was determined using the value of Δε of 15,200 M^−1^ cm^−1^ obtained above with BlaC at 486 nm (Fig. 2). In these experiments, data were acquired between 1 min and 14 hours due to the lag time required to charge an automatic sample changer. Thus, Ldt_fm_ was fully acylated at the beginning of the recording of the data (see above, Fig. 3A). The initial absorbance was subtracted to generate the kinetics presented in Fig. 3C. In these conditions, the increase in absorbance was due to enzyme turnover resulting in nitrocefin hydrolysis (Scheme 1 in Fig. 3). The observed turnover (5.29 ± 0.05 × 10^−5^ s^−1^) corresponds to the value of the kinetic parameter *k*_3_ since the bulk of Ldt_fm_ is acylated under these conditions (Fig. 3A). The value of *k*_3_ implies a half-life of 218 min for the acyl-enzyme. The high efficacy of acylation coupled to the low value of *k*_3_ accounts for the steady-state presence of EN* as the main form of Ldt_fm_ in the presence of nitrocefin.

### Competitive acylation of Ldt_fm_ by nitrocefin and the carbapenem imipenem

**Figure 4:**
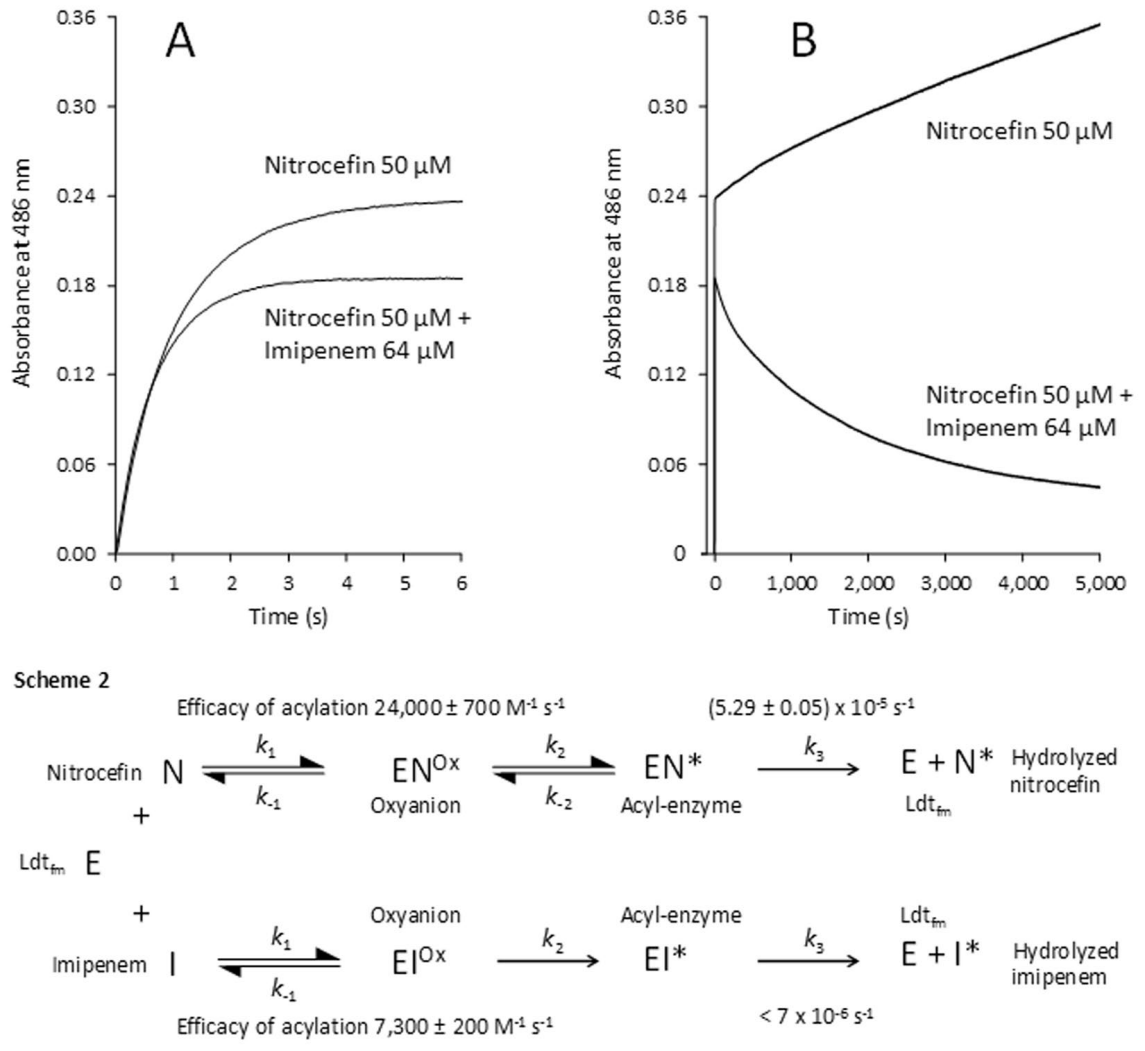
Competitive acylation of Ldt_fm_ by nitrocefin and imipenem. One syringe of the stopped-flow apparatus contained nitrocefin (100 µM) and imipenem (128 µM) in 100 mM sodium phosphate buffer (pH 6.0). The second syringe contained Ldt_fm_ (44 µM) in the same buffer. Equal volumes from syringes 1 and 2 were injected into the cuvette of the spectrophotometer and the absorbance was recorded at 486 nm. Concentrations indicated in the Figure take into account the two-fold dilution. Panels A and B represent the same kinetics for two timescales (0–6 s and 0–5000 s, respectively). In panel a, the increase in the absorbance at 486 nm mainly results from the rupture of the β-lactam ring of nitrocefin upon acylation of Ldt_fm_. In panel B, further increase in the absorbance observed in the absence of imipenem (upper curve) results from slow hydrolysis of nitrocefin. In the presence of imipenem, recyclization of the β-lactam ring of nitrocefin accounts for the decrease in the absorbance.

Ldt_fm_ (22 µM) was simultaneously exposed to nitrocefin (50 µM) and imipenem (64 µM) (Fig. 4A). The kinetics without imipenem was determined as a control. Imipenem had little impact on the initial rate of acylation of Ldt_fm_ by nitrocefin, as determined by the increase in the absorbance at 486 nm. Thus, imipenem did not significantly interfere with acylation of Ldt_fm_ by nitrocefin. Full acylation of Ldt_fm_ by nitrocefin was observed in *ca*. 3 s but a longer incubation in the presence of imipenem resulted in a decrease in the absorbance at 486 nm (Fig. 4B). This decrease could not be attributed to the hydrolysis of the acyl-enzyme that would result in an increase rather than a decrease in the absorbance of 486 nm. At this wavelength, reactions involving hydrolysis of imipenem or acylation of Ldt_fm_ by this drug had no impact on the absorbance (data not shown). We therefore concluded that native nitrocefin and nitrocefin bound to the catalytic Cys of Ldt_fm_ in the acyl-enzyme were in equilibrium and that imipenem displaced this equilibrium in favor of the native drug. In the competing acylation step, Ldt_fm_ was initially fully acylated by nitrocefin but imipenem gradually replaced nitrocefin in the enzyme active site since the acylation reaction was irreversible with imipenem but reversible with nitrocefin (Scheme 2). In the control experiment without imipenem, slow hydrolysis of nitrocefin was observed as expected from the kinetics displayed in Fig. 3C.

In the above experiments involving incubation of Ldt_fm_ with imipenem and nitrocefin, the recyclization reaction generates the native form of Ldt_fm_, which is expected to be competitively acylated by imipenem and nitrocefin. Recyclization followed by acylation with nitrocefin contributes to a futile cycle with no net impact on the concentration of nitrocefin. This implies that the net rate of nitrocefin recyclization should increase with the concentration of the competing imipenem. Testing recyclization of nitrocefin (50 µM) in the presence of various concentrations of imipenem (64, 128, 256, and 512 µM) revealed the expected increase in the net rate of nitrocefin recyclization (Supplementary Fig. 1).

### Competitive acylation of two forms of Ldt_fm_ by nitrocefin

**Figure 5:**
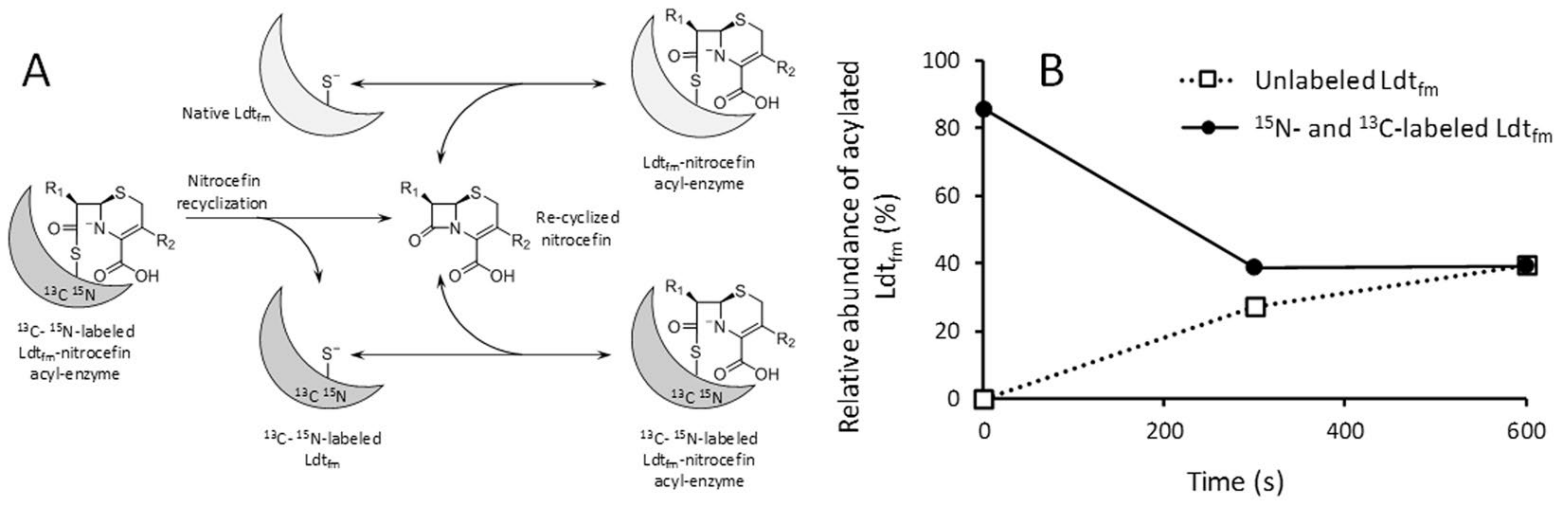
Detection of nitrocefin recyclization based on competitive acylation of unlabeled and labeled Ldt_fm_. (**A)** Schematic representation of the reactions. Resealing of the β-lactam ring of nitrocefin regenerates the native form of the drug (Re-cyclized nitrocefin) that leaves the Ldt_fm_ active site and competitively acylates Ldt_fm_ or^13^C-^15^N-labeled Ldt_fm_. (**B)** Uniformly^13^C- and^15^N-labeled Ldt_fm_ (2.2 nmole) was incubated with nitrocefin (1.5 nmole) for 1 min at 20 °C in 100 µl of 5 mM sodium phosphate buffer pH 6.0. The spectrum of the protein revealed *ca*. 82% acylation (time = 0). Unlabeled Ldt_fm_ (2.2 nmole in 1.46 µl) was added and mass spectra were recorded at 300 s and 600 s. The relative abundance of the acylated forms of Ldt_fm_ was deduced from the relative intensity of the peaks.

Since the reversibility of the acylation reaction was unprecedented for any β-lactam, our next objective was to demonstrate the reversibility of the acylation of Ldt_fm_ by nitrocefin using a second independent method. We surmise that resealing of the β-lactam ring of nitrocefin should regenerate the native form of the drug that could leave the Ldt_fm_ active site, diffuse in the medium, and competitively acylate another molecule of Ldt_fm_ (Fig. 5A). To bring these reactions to light, we prepared uniformly^13^C- and^15^N-labeled Ldt_fm_. The unlabeled and labeled forms of Ldt_fm_, which were readily identified by mass spectrometry, were expected to have similar catalytic properties since they only differ by their isotopic content. To test for the reversibility of the acylation reaction, a limiting amount of nitrocefin (1.5 nmole) was first incubated with labeled Ldt_fm_ (2.2 nmole) in 100 µl of 5 mM sodium phosphate buffer (pH 6.0). The extent of acylation (*ca*. 82%) indicated that the bulk of nitrocefin had reacted with Ldt_fm_ (Fig. 5B). Then, unlabeled Ldt_fm_ (2.2 nmole) was added and the partition of nitrocefin between the two forms of Ldt_fm_ was determined by mass spectrometry. After 600 s of incubation an equilibrium was reached and 40% of the labeled and unlabeled forms of Ldt_fm_ were acylated by nitrocefin. This result indicates that the β-lactam ring of nitrocefin was resealed within the active site of labeled Ldt_fm_ since this was the only source of native nitrocefin for acylation of unlabeled Ldt_fm_.

### Antibacterial activity of nitrocefin and imipenem resulting from inhibition of Ldt_fm_

We have previously reported activation of the L,D-transpeptidase pathway in a mutant of *Enterococcus faecium* (M512), which was selected in laboratory conditions on media containing increasing concentrations of ampicillin^11^. Depending upon the growth conditions, the peptidoglycan of the mutant M512 is exclusively cross-linked by the PBPs or by Ldt_fm_. In the presence of ampicillin (32 µg/ml), the D,D-transpeptidase activity of all PBPs produced by the mutant M512 is inhibited and Ldt_fm_ is the only functional transpeptidase. Antibiotic susceptibility testing in media containing ampicillin (32 µg/ml) was therefore used to assay for inhibition of Ldt_fm_ by nitrocefin. The broth microdilution assay revealed a 64-fold difference between the minimal inhibitory concentration (MIC) of nitrocefin (4 µg/ml) and that of imipenem (0.0625 µg/ml). Thus, nitrocefin was less active than imipenem in spite of a higher rate of acylation of Ldt_fm_.

## Discussion

In conclusion, we provide evidence for reversible acylation of the L,D-transpeptidase Ldt_fm_ by nitrocefin. To the best of our knowledge, this is the first time that reversible acylation is documented for a β-lactam antibiotic. This conclusion is based on two independent approaches. First, we show that the deacylation (recyclization) reaction regenerates the β-lactam ring of nitrocefin based on spectrophotometry, a technique that provided access to kinetic evaluation (Fig. 3). Second, we show that the deacylation reaction regenerates a native molecule of nitrocefin that eventually leaves the Ldt_fm_ active site and acylates another enzyme molecule. This was established by using two preparations of Ldt_fm_ that only differ by their isotopic content.

The irreversibility of the acylation step is generally attributed to the strain of the β-lactam ring although this has not yet been explored by computational investigations^15^. In agreement, compounds containing a γ-lactam ring reversibly react with nucleophilic serine enzymes. For example, recyclization has been extensively documented for the 5-membered cyclic urea group of avibactam^16, 17^, a β-lactamase inhibitor recently approved for clinical use in combination with the β-lactam ceftazidime^18^.

Our experimental design provides direct evidence that acylation is reversible for certain β-lactams (*e*.*g*. nitrocefin) but not for others (*e*.*g*. imipenem) since simultaneous incubation of Ldt_fm_ with the two drugs led to the rapid formation of the Ldt_fm_-nitrocefin acyl-enzyme, which was then slowly replaced by the Ldt_fm_-imipenem acyl-enzyme. The basis for this difference remains unknown, but it is tempting to speculate that it involves the fate of the negative charge that develops on the β-lactam nitrogen upon rupture of the β-lactam ring (Fig. 1). The determination of the NMR structure of the ertapenem-Ldt_fm_ acyl-enzyme has shown that the nitrogen atom of this carbapenem is protonated^19^. In the case of nitrocefin, protonation of the nitrogen atom is not expected to be required for stabilization of the acyl-enzyme since the negative charge is delocalized by the very strong electron withdrawing power of the dinitrophenyl substituent. This could account for reversible acylation of Ldt_fm_ by nitrocefin but not by imipenem. Of note, resealing of the β-lactam ring is thermodynamically more favorable for L,D-transpeptidases than for classical PBPs since the thioester formed with cysteine-containing L,D-transpeptidases has a much higher potential than the corresponding ester formed with serine-containing β-lactamases and D,D-transpeptidases.

The efficacy of acylation of Ldt_fm_ by nitrocefin (24,000 ± 700 M^−1^ s^−1^) (Fig. 3) was higher than that observed for any carbapenem-L,D-transpeptidase combination in previous studies^14, 20–22^. The efficacy of acylation of Ldt_fm_ was found to be lower for cephalosporins such as ceftriaxone (50 ± 5 M^−1^ s^−1^) and cephalotin (8.3 ± 3.3 M^−1^ s^−1^) or penams such as ampicillin (8.7 ± 0.3 M^−1^ s^−1^)^14^. Since cephalotin and nitrocefin are two cephalosporins harboring the same side-chain at positionC^7^ (Fig. 1), the 2,900-fold difference in the acylation efficacy observed between the two drugs is due to the side-chain at position C^3^. The NMR structure of Ldt_fm_ acylated by ertapenem indicates that there is little interaction, if any, between the side-chain at C^3^ and the enzyme^23^. In agreement, the efficacy of acylation mainly varies with the β-lactam class (carbapenem, cephalosporin, or penam), with a lesser impact originating from differences in their side-chains^14, 21^. These observations suggest that the high efficacy of acylation of Ldt_fm_ by nitrocefin depends upon an effect of the side-chain on the reactivity of the β-lactam ring, as opposed to a direct interaction of the side-chain with the enzyme. This effect of the side-chain may involve the polarization of the carbon-nitrogen bond of the β-lactam ring of nitrocefin.

In spite of the unexpected high efficacy of acylation, the antibacterial activity of nitrocefin was less than that of imipenem. This observation suggests that the irreversibility of the acylation reaction, rather than its efficacy, is important for antibacterial activity. Optimum antibacterial activity may therefore involve an additional reaction step that follows the acylation and prevents its reversibility. For carbapenems, this additional reaction step could be the protonation of the nitrogen of the β-lactam ring, as discussed above^23^. For most cephalosporins, irreversibility could result from the loss of a leaving group in the C^3^ side-chain (Fig. 1)^14, 22^. Of note, other types of rearrangements are instrumental to the inhibition of β-lactamases by clavulanate^5^.

In summary, our study shows that the strain of the β-lactam ring is not sufficient to prevent recyclization following acylation of Ldt_fm_. Drug efficacy may in part rely on a secondary modification of the drug in the active site to prevent the reversibility of the acylation step. These observations illustrate the complexity of parameters that should be taken into account for the optimization of β-lactams.

## Methods

### Materials

Stock solutions (25 mg/ml) of nitrocefin (Calbiochem) were prepared in 100% DMSO and stored at −20 °C. Dilutions were extemporaneously prepared in water. Stock solutions of imipenem (provided by Merck) were prepared extemporaneously in water.

### Protein purification

The plasmid constructs for production of the catalytic domain of Ldt_fm_ (residues 341 to 466) and of a soluble fragment of the β-lactamase BlaC from *M*. *tuberculosis* (residues 39 to 306) have been previously described^14, 24^. BlaC and Ldt_fm_ were produced in *E*. *coli* BL21(DE3) cells grown in brain heart infusion broth (BHI)^14, 24^. Labeling of Ldt_fm_ was obtained in M9 minimal medium containing ^13^C glucose and ^15^N NH_4_Cl^14^. Ldt_fm_ and BlaC were purified from clarified lysates by affinity and size-exclusion chromatography in 100 mM sodium phosphate buffer (pH 6.4) and in 25 mM Tris-HCl (pH 7.5) containing 300 mM NaCl, respectively^24, 25^. The purified enzymes were concentrated by ultrafiltration (Amicon Ultra-4 centrifugal filter devices, Millipore) and stored at −65 °C in the same buffers.

### Spectrophotometry

All kinetics were performed at 20 °C in 100 mM sodium phosphate (pH 6.0) either in a spectrophotometer (Cary 100; Varian SA) coupled to a stopped-flow apparatus (RX-2000; Applied Photophysics) or in a spectrophotometer (Cary 300) equipped with an automatic sample changer (Varian SA).

### Mass spectrometry

The formation of Ldt_fm_-β-lactam adducts was tested by incubating Ldt_fm_ with β-lactams at 20 °C in 5 mM sodium phosphate buffer (pH 6.0). Five µl of acetonitrile and 1 µl of 1% formic acid were extemporaneously added, and the reaction mixture was directly injected into the mass spectrometer (Qstar Pulsar I; Applied Biosystem) at a flow rate of 0.05 ml/min (acetonitrile, 50%, water, 49.5%, and formic acid, 0.5%; per volume). Spectra were acquired in the positive mode, as previously described^11^.

### Antibiotic susceptibility testing

The microdilution assay was performed in BHI broth containing 32 µg/ml of ampicillin (Euromedex) in 96-well plates. Each well (200 µl) was inoculated with overnight cultures to obtain 5 × 10^5^ cfu/ml. Plates were incubated for 24 h at 37 °C and the minimal inhibitory concentration was defined as the minimal concentration that prevented visible growth.

## Electronic supplementary material

Supporting Information
