# Supplementary material for: Reversible inactivation of a peptidoglycan transpeptidase by a β-lactam antibiotic mediated by β-lactam-ring recyclization in the enzyme active site
Source: Sci Rep. 2017 Aug 22;7:9136. doi: 10.1038/s41598-017-09341-8 (PMC5567249; doi:10.1038/s41598-017-09341-8)
Supplement: Supplementary file 1 — Supporting Information [file 41598_2017_9341_MOESM1_ESM.doc]

**Supplementary Information for**

**Reversible inactivation of a peptidoglycan transpeptidase by a β-lactam antibiotic mediated by β-lactam-ring recyclization in the enzyme active site**

Zainab Edoo, Michel Arthur, and Jean-Emmanuel Hugonnet

**Supplementary Fig. 1** Dependence of the rate of nitrocefin recyclization on the concentration of imipenem. One syringe of the stopped-flow apparatus contained nitrocefin (100 µM) and imipenem (128, 256, 512, or 1,024 µM) in 100 mM sodium phosphate buffer (pH 6.0). The second syringe contained Ldt_fm_ (30 µM) in the same buffer. Equal volumes from syringes 1 and 2 were injected into the cuvette of the spectrophotometer and the absorbance was recorded at 486 nm. Concentrations indicated in the figure take into account the two-fold dilution. The net rate of formation of native nitrocefin increased with the concentration of imipenem since imipenem and nitrocefin act in competition for the acylation of native Ldt_fm_ generated by the recyclization reaction.
